# Investigation of urban birds as source of β-lactamase-producing Gram-negative bacteria in Marseille city, France

**DOI:** 10.1186/s13028-019-0486-9

**Published:** 2019-10-31

**Authors:** Edgarthe Priscilla Ngaiganam, Isabelle Pagnier, Wafaa Chaalal, Thongpan Leangapichart, Selma Chabou, Jean-Marc Rolain, Seydina Mouhamadou Diene

**Affiliations:** 1 IRD, APHM, MEPHI, IHU-Mediterranee Infection, Aix-Marseille Univ, 19-21 bd Jean Moulin, 13005 Marseille, France; 2 IHU-Mediterranee Infection, Marseille, France

**Keywords:** Chickens, Extended-spectrum beta-lactamases (ESBL), Marseille, Pigeons, Urban birds, Yellow-legged gulls

## Abstract

**Background:**

We investigate here the presence of multidrug-resistant bacteria isolated from stool samples of yellow-legged gulls and chickens (n = 136) in urban parks and beaches of Marseille, France. Bacterial isolation was performed on selective media, including MacConkey agar with ceftriaxone and LBJMR medium. Antibiotic resistance genes, including extended-spectrum β-lactamases (ESBL) (i.e. *bla*_CTX-M_, *bla*_TEM_ and *bla*_SHV_), carbapenemases (*bla*_KPC_, *bla*_VIM_, *bla*_NDM_, *bla*_OXA-23_, *bla*_OXA-24_, *bla*_OXA-48_ and *bla*_OXA-58_) and colistin resistance genes (*mcr*-1 to *mcr*-5) were screened by real-time PCR and standard PCR and sequenced when found.

**Results:**

Of the 136 stools samples collected, seven ESBL-producing Gram-negative bacteria (BGN) and 12 colistin-resistant *Enterobacteriaceae* were isolated. Among them, five ESBL-producing *Escherichia coli* and eight colistin-resistant *Hafnia alvei* strains were identified. Four *bla*_TEM-1_ genes were detected in yellow-legged gulls and chickens. Three CTX-M-15 genes were detected in yellow-legged gulls and pigeons, and one CTX-M-1 in a yellow-legged gull. No *mcr*-1 to *mcr*-5 gene were detected in colistin-resistant isolates. Genotyping of *E. coli* strains revealed four different sequence types already described in humans and animals and one new sequence type.

**Conclusions:**

Urban birds, which are believed to have no contact with antibiotics appear as potential source of ESBL genes. Our findings highlight the important role of urban birds in the proliferation of multidrug-resistant bacteria and also the possible zoonotic transmission of such bacteria from wild birds to humans.

## Background

Extended-spectrum beta-lactamases (ESBLs) are mainly plasmid-encoded enzymes that confer resistance to beta-lactams, including broad spectrum cephalosporins. They can be transmitted between bacteria by horizontal gene transfer (HGT) via mobile genetic elements such as recombinant plasmids, transposons or integrons [1]. These ESBL enzymes mainly include class A β-lactamases, such as TEM, SHV and CTX-M type, that confer resistance to penicillin and cephalosporin classes. However, CTX-M β-lactamase enzymes are the most emerged and reported worldwide [1]. ESBL-producing *Enterobacteriaceae* cause infections in both humans and animals through contaminations of water and food [2]. Over the past decade, a high prevalence of antibiotic resistance genes has been reported and has stimulated the search for multidrug-resistant bacteria in poultry, pigeons [3, 4] and birds in different countries [5]. In recent years, several reports indicated the presence of ESBL-producing bacteria among different species of urban and wild birds with no apparent exposure to antimicrobial drugs [6]. As reported by Bonnedahl et al. [7], 9.4% of yellow-legged gulls (*Larus michahellis*) carried CTX-M type ESBL-producing *E. coli* in the South of France. Recently, Stedt et al. [8] have reported high and alarming levels of CTX-M genes in species of gulls in Europe and indicated that the ability of these birds to reach several countries could contribute to the spread of ESBLs. Therefore, pigeons and gulls, considered as migratory birds, have been pointed out as potential reservoirs and vectors for multidrug-resistant bacteria [8–10], especially gulls that are free-living aquatic birds. More recently, Wang et al. [11] have reported the role of wild birds in the global transmission of ESBL, AmpC β-lactamase, carbapenemase, and colistin resistance genes in *Enterobacteriaceae.* Because of their frequent movement in landfills and wastewater and the ease with which they collect food from various places, they can contribute to fecal contamination of natural water reservoirs or the food they come in contact with [5, 12]. Face to this phenomenon, we aim here to investigate and to characterize β-lactamase-producing bacteria and colistin-resistant bacteria isolated from urban birds in the city of Marseille, France.

## Methods

### Sample collection

**Fig. 1:**
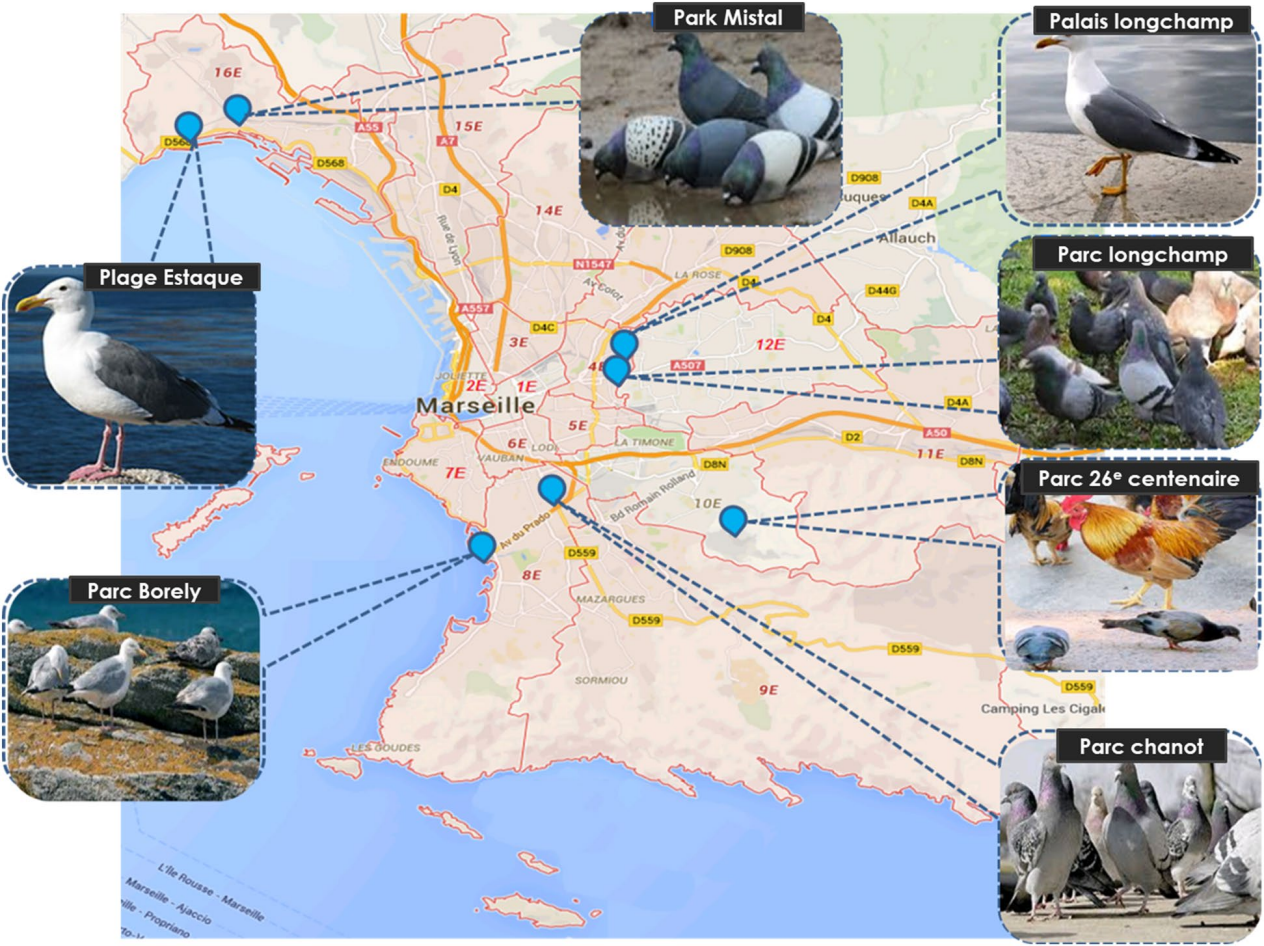
Map depicting sites of sample collection in Marseille, France. The sampling sites are located in different districts of the city of Marseille. The pigeon’s site, Longchamp Park and the site of yellow-legged gull Palais Longchamp, are both located in the 4th district. Parc chanot, site of pigeons and Parc Borely, site of yellow-legged gull is in the 8th district. The Parc du 26ème Centenaire, site of Pigeons and Chicken, is located in the 10th arrondissement. Finally, the Park Mistral (Espace Mistral), site of pigeons and Beach of Estaque (Plages de Corbière), site of yellow-legged gull, are located in the 16th district

Between July and September 2016, six parks located respectively in four different districts of Marseille city, and one beach located in the 16th district of Marseille, were investigated (Fig. 1 and Additional file 1). A total of 136 stool samples from 71 pigeons (*Columba livia f. urbana*), 28 chickens (*Gallus gallus domesticus*), and 37 yellow-legged gulls (*Larus michahellis*), were collected (Fig. 1 and Additional file 1). About 10 g of feces per sample was collected from the ground using sterile forceps and transferred into sterile 1.5 mL Eppendorf tubes. The samples were then immediately placed on ice during transport. Microbiological analyses were then carried the day after sampling.

### DNA extraction and ESBL detection

Genomic DNA from 136 feces samples was prepared from an overnight incubation at 56 °C and extracted with the EZ1 DNeasy Blood Tissue kit (Qiagen GmbH, Hilden, Germany) using the protocol of the manufacturer. The presence of genes encoding for ESBLs (CTX-M, TEM and SHV, PER, GES, and VEB), carbapenemases (KPC, VM, NDM, OXA-23, OXA-24, OXA-48 and OXA-58) and *mcr*-1 to *mcr*-*5* genes were investigated by real-time polymerase chain reaction (RT-PCR) with the specific primers as previously described [13–16]. Moreover, we designed here primers and probe for detection of All *bla*_TEM_ gene variants (ALLTEM_RT_F: 5′-TTCTGCTATGTGGTGCGGTA-3′; ALLTEM_RT_R: 3′-GTCCTCCGATCGTTGTCAGA-5′; ALLTEM_RT_Probe: 5′-AACTCGGTCGCCGCATACACTATTCTCAGA-3′).

### Isolation and identification of bacterial strains

To isolate β-lactamase-producing bacteria and colistin resistant bacteria, 1 g of feces samples was mixed with 1 mL sterile water and 100 µL were enriched in Tryptic Soy Broth (BBL™ Trypticase™ Soy Broth, Becton, Dickinson and Company Sparks, MD 21152, USA) and incubated overnight at 37 °C for preliminary analysis. RT-PCR targeting β-lactamase genes were used to screen all samples for positive ESBL genes. When a positive sample was suspected of isolates producing ESBL genes, bacteria were cultured on MacConkey agar medium with added ceftriaxone (2 µg/mL). On the other hand, in order to look for colistin resistant bacteria, all sample were directly cultured on the selective Lucie Bardet–Jean-Marc Rolain (LBJMR) medium [17] containing 4 µg/mL of colistin and 50 µg/mL of vancomycin. All cultures were then incubated at 37 °C for 24 h. From one to eight colonies of different morphologies were collected in each medium and reisolated on Trypticase Soy Agar (TSA, BioMerieux SA, France). Each of these colonies was subcultured 3 additional times to obtain a monoculture. Isolates were then identified by matrix assisted laser desorption/ionization, time of flight mass spectrometry (MALDI-TOF MS) and MALDI-Biotyper 3.0 software (Bruker Daltonics, Bremen, Germany) as described previously [18]. Bacteria are efficiently identified at the genus and species level when score values are ranging from 2.3 to 3.0 and at the secure genus identification and probable species identification when score values are ranged from 2.0 to 2.3 [18].

### Antimicrobial susceptibility testing

Antibiotic susceptibility testing (AST) was performed using the agar disk diffusion method. Bacterial strains (n = 19) isolated from birds were suspended in a saline solution to a density of 0.5 McFarland. The surface of Mueller–Hinton agar plates (Merck, Darmstadt, Germany) was inoculated and the antibiotic disks were placed. After aerobic incubation at 37 °C for 18–22 h, bacterial resistance was determined by measuring the inhibition zone according to the guidelines of CASSFM/EUCAST 2019 (v.1.0) (https://www.sfm-microbiologie.org/wp-content/uploads/2019/02/CASFM2019_V1.0.pdf). The following antibiotics were tested (µg/disk): amoxicillin (20), amoxicillin–clavulanic acid (20/10), cefepime (30), piperacillin–tazobactam (30/6), cefalotin (30), ceftriaxone (30), ertapenem (10), imipenem (10), fosfomycin (200), nitrofuran (100), trimethoprim–sulfamethoxazole (1.25/23.75), amikacin (30), ciprofloxacin (5), colistin (50), and gentamicin (10). For bacteria isolated on LBJMR medium, colistin MICs were determined by microdilution method using Mueller–Hinton (MH) liquid medium.

### Sequence analysis and MLST

Aminoglycoside resistance genes (i.e. *aad, ant, aph, aac*(*6′*)-*Ib, aac*(*3′*), *armA*, and *aac*(*6′*)-*Ib*-*cr*) were investigated in *Escherichia coli* isolate (B32 strain) resistant to gentamicin and doxycycline. On overall, by qPCR and standard PCR we investigated genes encoding for extend-spectrum-β-lactamases (*bla*_SHV_, *bla*_VEB_*, bla*_GES_, *bla*_PER_), carbapenemases (VIM, NDM, OXA-23, OXA-24, OXA-48, OXA-58), and mobile colistin resistance genes (*mcr*-*1* to *mcr*-*5*) using primers and probes previously reported [19].

All positive PCR products for genes encoding for ESBL that conferred resistance to 3rd generation cephalosporins were purified using the QIAquick PCR purification kit in concordance with the manufacturer’s instructions (Qiagen, Hilden, Germany). Sequencing was performed using the BigDye Terminator Cycle Sequencing kit and an automated fluorescent DNA sequencer ABI 3730xl (Applied Biosystems, Foster City, CA, USA) in accordance with the manufacturer’s instructions. The nucleotide sequences obtained were compared with those from the NCBI database by BlastN analysis. Multilocus sequence typing (MLST) was performed for five ESBL-producing *E. coli* isolates using genes from both the Warwick (*adk*, *fumC*, *gyrB*, *icd*, *mdh*, *purA*, and *recA*) (http://mlst.warwick.ac.uk/mlst/dbs/Ecoli) and Pasteur Institute (*icd*, *pabB*, *polB*, *putP*, *trpA*, and *trpB*) (http://www.pasteur.fr/recherche/genopole/PF8/mlst/) schemes. The ST not found in the database was submitted to the Pasteur MLST database to define the new profile.

## Results

From the 136 stool samples from 71 pigeons (*Columba livia f. urbana*), 28 chickens (*Gallus gallus domesticus*), and 37 yellow-legged gulls (*Larus michahellis*), 15 samples were positive by the RT-PCR targeting ESBL-encoding genes. The culture of these samples on the MacConkey with ceftriaxone medium reveals seven isolates from six samples (Table 1). On the selective LBJMR medium, the culture results to 12 isolates from 12 samples (Table 1). From the 19 isolated strains, bacterial identification by MALDI-TOF revealed, with a score ranging between 2.15 and 2.95, the presence of *E. coli* (n = 5), *Cronobacter sakazakii* (n = 1), *Pseudomonas aeruginosa* (n = 1), *Hafnia alvei* (n = 8), *Proteus hauseri* (n = 1)*, Panteoa ananatis* (n = 1), *Providencia alcalifaciens* (n = 1) and *Serratia marcescens* (n = 1).

**Table 1:** General features of all bacterial strains isolated from urban birds from the different investigated sites of Marseille city

Strain name	Isolates	Sources	Sites	Antibiotic resistance phenotype	MIC_CT_ (µg/ml)	*bla*_CTX-M_ gene^a^	*bla*_TEM_ gene^a^	MLST
Bacterial strains isolated from the MacConkey agar								
B11	*Escherichia coli*	Yellow-legged gull	Site 1	AX, AMC, CRO, FEP, STX, CIP	0.25	CTX-M-15	–	ST10
B32	*Escherichia coli*	Chicken	Site 2	AX, AMC, CRO, FEP, STX, CN	0.25	–	TEM-1	ST1652
B60	*Escherichia coli*	Yellow-legged gull	Site 3	AX, AMC, CRO, FEP, STX	0.25	CTX-M-15	–	ST38
A61	*Escherichia coli*	Pigeon	Site 2	AX, KF, CRO, FEP	0.25	CTX-M-15	–	ST34
B62	*Escherichia coli*	Yellow-legged gull	Site 3	AX, AMC, CRO, FEP	0.25	CTX-M-1	TEM-1	ST857
B62	*Cronobacter sakazakii*	Yellow-legged gull	Site 3	AX, AMC, CRO, FF	0.25	–	TEM-1	–
B34	*Pseudomonas aeruginosa*	Chicken	Site 2	AX, KF, CRO, F, STX	0.25	–	TEM-1	–
Bacterial strains isolated from the LBJMR agar								
B42	*Hafnia alvei*	Chicken	Site 2	AX, AMC, KF, CT	8	–	–	–
B02	*Hafnia alvei*	Yellow-legged gull	Site 1	AX, AMC, KF, CT	4	–	–	–
B04	*Hafnia alvei*	Yellow-legged gull	Site 1	KF, CT	4	–	–	–
B11	*Hafnia alvei*	Yellow-legged gull	Site 1	AX, AMC, KF, CT	4	–	–	–
B21	*Hafnia alvei*	Yellow-legged gull	Site 4	KF, CT	4	–	–	–
B47	*Hafnia alvei*	Chicken	Site 2	AX, AMC, KF, CT	4	–	–	–
B59	*Hafnia alvei*	Yellow-legged gull	Site 3	AX, AMC, KF, FF, F, CT	4	–	–	–
A63	*Hafnia alvei*	Pigeon	Site 2	AX, AMC, KF, FF, F, CT	4	–	–	–
A61	*Panteoa ananatis*	Pigeon	Site 2	AX, AMC, KF, FF, F, CT	> 256	–	–	–
B05	*Proteus hauseri*	Yellow-legged gull	Site 1	F, CT	> 256	–	–	–
B22	*Serratia marcescens*	Yellow-legged gull	Site 4	F, CT	> 256	–	–	–
B36	*Providencia alcalifaciens*	Chicken	Site 2	F, CT	> 256	–	–	–

Site 1: Palais Longchamp; Site 2: Parc 26e Centenaire; Site 3: Plage Estaque; Site 4 : Parc Borely

*AX* amoxicillin, *AMC* amoxicillin–clavulanic acid, *KF* cefalotin, *CRO* ceftriaxone, *FEP* cefepime, *ERT* ertapenem, *IMP* imipenem, *FF* fosfomycin, *F* nitrofurantoin, *SXT* trimethoprim–sulfamethoxazole, *CIP* ciprofloxacin, *CT* colistin, *CN* gentamycin, *ST* sequence type

^a^Obtained β-lactamase sequences were compared with reference protein sequences of bets hit sequences which were *bla*_CTX-M-15_ (JQ686199); *bla*_CTX-M-1_ (X92506); and *bla*_TEM-1_ (JQ735917) (cf. Additional file 2)

Only bacterial strains isolated on the MacConkey agar exhibited reduced susceptibility to 3rd generation cephalosporin (ceftriaxone) and only *E. coli* strains were resistant to cefepime (4th generation cephalosporin). Resistance also to trimethoprim–sulfamethoxazole, ciprofloxacin, doxycycline, and gentamicin was observed in some of these isolates (Table 1). However, all these strains from this MacConkey agar remained susceptible to carbapenems and colistin. For 3rd generation cephalosporin-resistant isolates, the standard PCR performed and sequencing revealed the presence of ESBL genes such as *bla*_CTX-M_ and/or *bla*_TEM_ with amino acid similarity ranging from 99.61 to 100% (Additional file 1). Four genes encoding two CTX-M variants were identified (Table 1). The *bla*_CTX-M-15_ gene was found in three *E. coli* isolates from two yellow-legged gulls and one pigeon. Only one *E. coli* isolate from a yellow-legged gull harboured simultaneously *bla*_CTX-M-1_ and *bla*_TEM-1_. Moreover, four *bla*_TEM-1_ β-lactamase genes were detected in two *E. coli*, one *C. sakazakii* and one *P. aeruginosa* isolates from chicken and yellow-legged gulls (Table 1). Co-presence of ESBL-producing strains e.g. *C. sakazakii* and *E. coli* was observed in a yellow-legged gull sample. These latter were resistant to amoxicillin; amoxicillin–clavulanic acid, cefalotin, ceftriaxone and were positive for the *bla*_TEM-1_ gene. One of the *E. coli* isolate (B32 strain) carrying *bla*_TEM-1_ was resistant to gentamicin and doxycycline, but none of the seven aminoglycoside resistance genes investigated were detected in this isolate. Besides, as expected, all strains isolated on the LBJMR agar were resistant to colistin (MIC ≥ 4 μg/mL) and included bacteria naturally resistant to colistin (Table 1). Resistance to β-lactams including amoxicillin, amoxicillin-acid clavulanic, and cephalothin was observed in most of *H. alvei* strains. Interestingly, for two *H. alvei* strains, an atypical resistance phenotype was observed, i.e. they were resistant to cephalothin but susceptible to amoxicillin (Table 1). All positive qPCR for targeted genes from samples were confirmed by standard PCR from isolated strains by culture of the corresponded sample. All these isolates were susceptible to carbapenems, amikacin, trimethoprim–sulfamethoxazole, ciprofloxacin, doxycycline, and gentamycin. Overall, qPCR and standard PCR searches for resistance genes encoding for *bla*_SHV_, *bla*_VEB_, *bla*_GES_, *bla*_PER_, carbapenemases, and mobile colistin resistance genes were negative for all isolates.

Genotyping by MLST was performed for *E. coli* isolates and revealed five different sequence types (STs) including ST10, ST34, ST38, ST1652 and a new ST which has been defined as ST857. The *E. coli* ST10 and ST38 carried the *bla*_CTX-M-15_ gene and were from yellow-legged gulls, whereas the *E. coli* ST34 belonging to the ST10 complex was isolated from a pigeon and also harbored a *bla*_CTX-M-15_ gene. Finally, *E. coli* ST1652, isolated from a chicken, carried the *bla*_TEM-1_ gene and the *E. coli* ST-new carried *bla*_CTX-M-1_ and was from yellow-legged gull.

## Discussion

In this study, we report CTX-M and TEM β-lactamases in Gram-negative bacterial isolates from urban birds in Marseille. Unlike previous studies reporting a high prevalence of ESBL-producing bacteria in gulls [11, 20, 21] and chickens [3, 22, 23], we observe here, a low prevalence (1.47%) of ESBL-producing bacteria in chickens. This is probably due to that they are domestic chickens that have never received food additives or growth factors and live only in non-livestock parks. Here, we isolated a β-lactamase-producing *P. aeruginosa* from a chicken which was susceptible to amoxicillin–clavulanic acid and harbored the *bla*_TEM-1_ gene only. With respect to the literature, *P. aeruginosa* is considered as one of the leading causes of infections in livestock and companion animals and *P. aeruginosa* isolates producing ESBL genes, such as *bla*_PER-1_, *bla*_CTX-M_, *bla*_SHV_ and *bla*_TEM_ were reported in camel meat with a high prevalence [24]. Moreover, ESBL-producing *P. aeruginosa* isolates have also been observed from clinical settings, poultry, cattle and vegetables [25].

Interestingly, there was a simultaneous presence of ESBL-producing *C. sakazakii* and *E. coli* in a yellow-legged gull. In 2015, a study conducted by Zurfluh et al. [26] reported the presence of *C. sakazakii* producing ESBL SHV-2 type in fresh vegetables imported to Switzerland from the Dominican Republic, India, Thailand and Vietnam. It should be noted that *C. sakazakii* is an opportunistic Gram-negative, pathogenic bacterium with no spore formation, sometimes associated with sporadic cases or brief outbreaks of sepsis, meningitis, encephalitis, necrotizing enterocolitis and bacteremia in adults and newborns preterm neonates [27]. In many cases, the reservoir of *C. sakazakii* is unknown, and there are increasing reports that powdered infant formula is the source of *C. sakazakii* infections [28]. Our isolate was resistant to cephalothin as described by Xu et al. [29] who reported *Cronobacter* spp. isolates resistant to cephalothin in ready-to-eat foods and the potential risk of consumer contamination.

*Hafnia alvei*, an anaerobic Gram-negative bacterium, was isolated from all species birds investigated here. They are often present in the environment and a part of the animal gut [30]. This bacterium is rarely isolated from humans but can sometimes be an opportunistic pathogen responsible for nosocomial infections [30, 31]. All *H. alvei* isolated from birds in this study were resistant to cephalothin and colistin, which is a natural resistance [31]. However, most of these isolates were also resistant to amoxicillin and amoxicillin–clavulanic acid but susceptible to ceftriaxone, which is similar to a study conducted by Angela et al. [32] on broad spectrum cephalosporin-resistant *Enterobacteriaceae* isolated from humans.

Except the ST857 new sequence type identified here, the ST10, ST38, ST34 and ST1652 ESBL-producing *E. coli* described here are widely reported in humans, animals and in the environment (waters and wild birds) [6, 33–35]. Therefore, our study is consistent with previous studies in which human and animal isolates shared mainly identical STs, suggesting a possible transmission. In France, very few studies have been conducted on urban birds and our study is the first to investigate antibiotic-resistant bacteria in parks and beaches. We observed that ESBL-producing *Enterobacteriaceae* were more frequently isolated from beach gulls than chickens and pigeons as reported by Stedt et al. [8]. They reported that the ESBL-producing bacteria were frequently detected in gulls (906 ESBLs from a collection of 3158 samples, 28.7%) with significant variations in prevalence rates between countries. We also observed that the STs isolated in our study were completely different from the STs found in yellow-legged gull in the other sites of south of France [7]. Pets, such as dogs and cats, are potential sources of transmission of antibiotic-resistant bacteria due to their close contact with humans and the intensive use of antimicrobial agents in humans and in the domestic animals. It should be noted that parks are open to dogs that are suspected of being a source of resistance genes. Indeed, the spread of resistance can occur directly or indirectly, through feeding, water and animal waste disposal in farm fields. Therefore, our study supports the idea that the beaches can be considered as sources of antibiotic-resistant bacteria.

## Conclusion

Urban birds can be vectors for the transmission of ESBL-producing bacteria between human and animals. It may be due to their close contact with human activities, in addition to the role of parks and beaches as a meeting place for domestic and human animals, urban birds and environmental waters. To the best of our knowledge, we report here, for the first time, the presence of antibiotic-resistant bacteria from birds, especially yellow-legged gulls and chickens, in Marseille’s parks and beaches. Further works such as whole genome sequencing of such bacteria from birds of different regions would be interesting to investigate how such bacteria are circulating via urban birds.

## Supplementary information

**Additional file 1.** Protein alignment of identified β-lactamase enzymes from bird samples. **A)** Sequence alignment of TEM-1 proteins compared with reference TEM-1 protein (JQ735917) retrieved from the ARG-ANNOT database. **B)** Sequence alignment of CTX-M proteins compared with reference proteins of CTX-M-15 (JQ686199) and CTX-M-1 (X92506). All sequenced *bla*_TEM-1_ genes (814-bp) exhibited 99.61% aa similarity with the TEM-1 reference sequence. *bla*_CTX-M-15_ and *bla*_CTX-M-1_ genes (855-bp) exhibited 100% aa similarity with CTX-M-15 and CTX-M-1 respectively.

**Additional file 2.** Sampling details according to the investigated locations.

## Data Availability

Not applicable
