# Supplementary figures and images for: Investigation of urban birds as source of β-lactamase-producing Gram-negative bacteria in Marseille city, France
Source: Acta Vet Scand. 2019 Oct 31;61:51. doi: 10.1186/s13028-019-0486-9 (PMC6822345; doi:10.1186/s13028-019-0486-9)

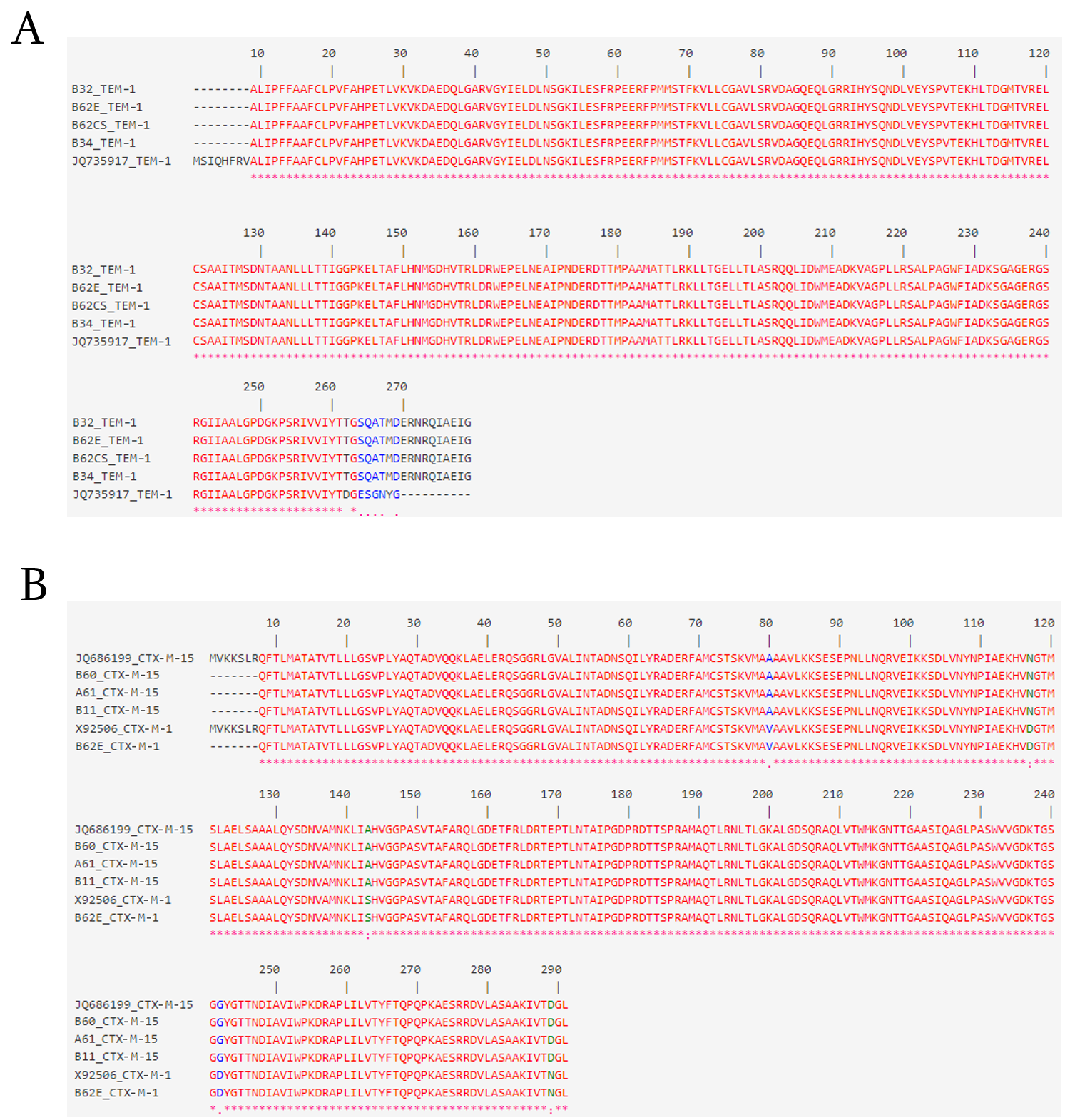

Supplement: Supplementary file 1 — Additional file 1. Protein alignment of identified β-lactamase enzymes from bird samples. A) Sequence alignment of TEM-1 proteins compared with reference TEM-1 protein (JQ735917) retrieved from the ARG-ANNOT database. B) Sequence alignment of CTX-M proteins compared with reference proteins of CTX-M-15 (JQ686199) and CTX-M-1 (X92506). All sequenced blaTEM-1 genes (814-bp) exhibited 99.61% aa similarity with the TEM-1 reference sequence. blaCTX-M-15 and blaCTX-M-1 genes (855-bp) exhibited 100% aa similarity with CTX-M-15 and CTX-M-1 respectively. [file 13028_2019_486_MOESM1_ESM.tif]
