# Supplementary material for: Investigation of urban birds as source of β-lactamase-producing Gram-negative bacteria in Marseille city, France
Source: Acta Vet Scand. 2019 Oct 31;61:51. doi: 10.1186/s13028-019-0486-9 (PMC6822345; doi:10.1186/s13028-019-0486-9)
Supplement: Supplementary file 2 — Additional file 2. Sampling details according to the investigated locations. [file 13028_2019_486_MOESM2_ESM.doc]

**Additional file 2**: Sampling details according to the investigated locations.

|  | Number of samples | Parc  Chanot | Parc  Longchamp | Parc 26^e^  Centenaire | Parc  Mistral | Palais  Longchamp | Parc  Borely | Plage  Estaque | Positive samples in qPCR assay  (n=15) | Positive samples in culture  (n=6) |
| --- | --- | --- | --- | --- | --- | --- | --- | --- | --- | --- |
| **Pigeon** | 71 | 17 | 26 | 24 | 4 |  |  |  | 2 | 1 |
| **Chicken** | 28 |  |  | 28 |  |  |  |  | 4 | 2 |
| **Yellow-legged gull** | 37 |  |  |  |  | 14 | 13 | 10 | 9 | 3 |
